# Supplementary material for: Breaking the deadlock: A study on the pathway and effects of reshaping the sustainable marketing capability of Chinese time-honored brands
Source: PLoS One. 2025 Jun 26;20(6):e0326329. doi: 10.1371/journal.pone.0326329 (PMC12200666; doi:10.1371/journal.pone.0326329)
Supplement: Attachment — Submitted filename: Response to Reviewers.docx [file pone.0326329.s002.docx]

**Response to Reviewers**

Manuscript ID: PONE-D-24-48512

Manuscript Title: Breaking the deadlock: a study on the pathway and effects of reshaping the sustainable marketing capability of Chinese time-honored brands

**Response to Editorial Requirements**

We thank the Academic Editor and the journal office for the opportunity to revise and resubmit our manuscript entitled "Breaking the deadlock: a study on the pathway and effects of reshaping the sustainable marketing capability of Chinese time-honored brands" (Manuscript ID: PONE-D-24-48512).

In accordance with the submission guidelines, we have completed the following:

1. We have uploaded a revised manuscript with tracked changes to highlight all modifications made, as well as a clean version of the manuscript without tracked changes for the final evaluation.
2. We have provided all relevant data required to replicate the results of our study, including the raw values and summary statistics. Since our study is based on a quantitative survey design and does not involve laboratory-based experimental procedures, it is not applicable to upload experimental protocols to protocols.io. However, we have ensured the reproducibility of our results by fully sharing the dataset and measurement instruments used in our analysis.
3. No changes were made to the financial disclosure statement. The original disclosure remains accurate and up-to-date.
4. To enhance transparency and facilitate editorial review, we have color-coded all revisions in the manuscript according to their source. Specifically, responses to the Academic Editor and journal requirements are marked in **blue font**, revisions addressing Reviewer #1’s comments are marked in **purple font**, and those responding to Reviewer #2 are marked in **red font**. In addition, changes made by the authors upon internal post-revision review—such as clarifications or minor corrections not directly prompted by reviewers—are marked in **dark red** font. These changes can be found in the file titled “Revised Manuscript with Track Changes”.

Below, we provide a point-by-point response to the editor’s and each of the reviewers’ comments, with the corresponding revisions clearly marked in the revised manuscript.

**Response to Journal Requirement**

**Editor Comment #1:**

Please ensure that your manuscript meets PLOS ONE's style requirements, including those for file naming.

**Response:**

We thank you for this reminder. We have carefully reviewed the PLOS ONE formatting guidelines and revised the manuscript accordingly to ensure compliance. Specifically, we adjusted the structure, section headings, figure referencing, reference format, and English grammar to align with the PLOS ONE style templates. We have also renamed our files according to the journal’s file-naming conventions.

**Editor Comment #2:**

Please provide additional details regarding the participant consent. In the ethics statement in the Methods and online submission information, please ensure that you have specified the following:

(1) whether consent was informed and

(2) what type you obtained (for instance, written or verbal, and if verbal, how it was documented and witnessed). If your study included minors, state whether you obtained consent from parents or guardians.

**Response:**

We appreciate this important comment. We have now added a complete ethics statement to the Methodology section of the manuscript (see 3.2 Ethics Statement, which is located from line 447 to line 453, on page 18-19 of the manuscript). Specifically, we confirmed that informed verbal consent was obtained from all participants prior to survey administration. Participants were clearly informed about the study’s objectives, procedures, and voluntary nature, and they provided verbal consent before proceeding. No minors were involved in this study. The consent process was reviewed and approved by the Ethics Committee of Chuxiong Normal University.

Additionally, we confirm that this manuscript does not involve any retrospective studies of medical records or archived biological samples.

**Editor Comment #3:**

Please confirm at this time whether or not your submission contains all raw data required to replicate the results of your study. Authors must share the “minimal data set” for their submission. If your submission does not contain these data, please either upload them as Supporting Information files or deposit them to a stable, public repository and provide us with the relevant URLs, DOIs, or accession numbers.

**Response:**

We thank you for highlighting this important requirement. We confirm that we have provided all the data necessary to replicate the findings of our study. This includes the raw scores underlying the descriptive statistics, inferential tests, and structural equation modeling. These data have been uploaded as Supporting Information: [original data.xlsx and original data.sav]. The data were fully anonymized prior to sharing, and no personally identifiable information was included.

**Editor Comment #4:**

When completing the data availability statement of the submission form, you indicated that you will make your data available on acceptance. We strongly recommend all authors decide on a data sharing plan before acceptance. Please note that your entire data will need to be made freely accessible if your manuscript is accepted for publication.

**Response:**

We fully support ’s open data policy of PLOS ONE. We confirm that we have now prepared a complete data-sharing plan and agree to make all relevant data freely accessible upon publication. The minimal dataset necessary to replicate the findings of this study has been compiled and uploaded as Supporting Information. These files include raw and summary data and will be available without restrictions upon publication.

**Editor Comment #5:**

Please ensure that you refer to Figure 1 in your text, as, if accepted, production will need this reference to link the reader to the figure.

**Response:**

Thank you for this helpful reminder. We have now added explicit in-text references to Figure 1 in the manuscript. The reference appears on page 17, line 429.

**Comments to the Author**

**1. Is the manuscript technically sound, and do the data support the conclusions?**

Reviewer #1: Yes

Reviewer #2: Partly

**Response:**

We appreciate the reviewers’ assessment of the technical merits of our study. We would like to emphasize that we rigorously designed our methodology, ensured an adequate sample size, and implemented robust analytical procedures, such as structural equation modeling (SEM) and bootstrapping techniques. In response to Reviewer #2’s concerns, we have clarified the sampling logic, expanded the description of sample characteristics, and explicitly explained how key dimensions (e.g., MC, ML, MO) were derived. These improvements strengthen the link between the data and conclusions and reinforce the overall technical soundness of the manuscript.

**2. Has the statistical analysis been performed appropriately and rigorously?**

Reviewer #1: Yes

Reviewer #2: No

**Response:**

We thank the reviewers for their assessment of our statistical analyses. While Reviewer #1 acknowledged the rigor of our statistical analysis, we also recognize Reviewer #2’s concern and have taken additional steps to clarify and reinforce the methodological transparency of our study in the revised manuscript.

Specifically, we have elaborated on the design and validation of the questionnaire, including the origins of the measurement scales, translation process, and reliability and validity testing. Furthermore, we clarified the dimensional structure reported in Table 3, indicating that these dimensions were predefined based on the literature and validated through confirmatory factor analysis (CFA), not derived from exploratory factor analysis (EFA).

We also ensured that key statistical metrics, including Cronbach’s alpha, AVE, CR, factor loadings, discriminant validity, and model fit indices, were clearly reported and met the accepted thresholds. These enhancements aim to provide a more rigorous and transparent presentation of our analytical procedure.

**3. Have the authors made all data underlying the findings in their manuscript fully available?**

Reviewer #1: Yes

Reviewer #2: No

**Response:**

We thank the reviewers for highlighting the importance of data transparency in this study. In the revised submission, we fully addressed this concern by uploading the minimal data set required to replicate all statistical findings, including raw item responses, computed scale values, and key summary statistics.

All data were anonymized, with no personal identifiers retained, and conformed to ethical and privacy standards. We have also revised the Data Availability Statement in the manuscript and submission system to reflect full compliance with the PLOS ONE open data policy.

We believe that this satisfies the journal’s data availability requirements and supports research reproducibility.

**4. Is the manuscript presented in an intelligible fashion and written in standard English?**

Reviewer #1: Yes

Reviewer #2: No

**Response:**

We appreciate this observation and fully understand the importance of language clarity for international readers. In response, after completing all content-related revisions, we conducted a comprehensive round of American English editing and grammatical correction across the entire manuscript. Particular attention was paid to the consistency of terminology, clarity of conceptual explanations, and syntactic accuracy.

We believe that the current version presents the arguments and findings in an intelligible, coherent, and fluent manner. We are confident that the manuscript now meets the language and expression standards required for publication in PLOS ONE.

**Response to Reviewer Comments (General Statement for Item 5)**

We sincerely thank both the reviewers for their thoughtful and constructive feedback. We are pleased that Reviewer #1 recognized the strength and value of our study, and we also appreciate Reviewer #2’s detailed suggestions that helped us improve the clarity, methodological rigor, and overall presentation of this manuscript.

In this revised version, we have addressed all the comments point by point. The main revisions include the following:

- Clarifying key concepts and definitions to avoid confusion caused by abbreviations.
- Enhancing the methodological section by elaborating on the questionnaire design, sampling process, and variable construction.
- Expanding the conclusion to better address the research questions, theoretical contributions, and practical implications.
- The manuscript language was polished to improve clarity and intelligibility.
- Ensuring data availability and ethical compliance according to journal policy.

Furthermore, we affirm the following:

- This study has not been published previously and is not under consideration elsewhere.
- There are no concerns regarding research ethics, participant consent, or data collection integrity.
- There are no conflicts related to publication ethics, including plagiarism or redundant publications.

Below, we provide detailed responses to each reviewer’s comment, indicating the corresponding revisions made in the manuscript.

**Response to Reviewer**

**Reviewer #1 Comment:**

The article 'Breaking the deadlock: a study on the pathway and effects of reshaping the sustainable marketing capability of Chinese time-honored brands' presents a highly engaging and valuable contribution to the field. It was a pleasure to review this work.

The authors address a timely and significant topic, focusing on the sustainability of traditional brands in a rapidly evolving business environment. The theoretical model developed in this study is well-grounded, effectively integrating concepts of ambidextrous marketing, sustainable marketing capabilities, and corporate performance. This integration provides a comprehensive framework for understanding the challenges and opportunities faced by time-honored brands in China.

Methodologically, the research is robust, employing advanced statistical techniques, including structural equation modeling. This approach lends credibility to the findings and enhances the overall rigor of the study. Furthermore, the insights derived from this research offer valuable guidance for managers of traditional brands, potentially aiding in the development of more effective and sustainable marketing strategies.

While the study is strong in many aspects, there is room for improvement. Specifically, the paper would benefit from a more in-depth discussion of the particularities of the sample of brands studied. A more detailed exploration of the unique characteristics of these time-honored brands and their specific market contexts would enhance the generalizability and applicability of the findings.

**Response:**

We sincerely thank Reviewer 1 for the thoughtful and encouraging feedback on the theoretical design, methodological rigor, and practical implications of our study.

In response to your valuable suggestions regarding the discussion of sample characteristics and contextual uniqueness, we have made the following revisions:

- In the Introduction section, we added a new paragraph following the “Research Contributions” to explicitly highlight the regional and cultural embeddedness of the sample firms. This addition clarifies how the specific historical, structural, and market conditions of Southwest China shape the developmental challenges and opportunities faced by China Time-Honored Brands (see page 5, lines 120–126).
- In the newly added Discussion section (5.1 Sample Characteristics and Contextual Influence), we elaborate on the industry distribution, cultural attributes, innovation dynamics, and policy environment of the surveyed firms. We show how these factors interact with marketing exploration and exploitation capabilities and how they influence the sustainable development pathways for legacy brands (see page 33-34, lines 706–732).

These revisions aim to strengthen the contextual richness of the study and improve the generalizability and applicability of the findings, as you have insightfully suggested. We greatly appreciate your constructive feedback.

**Response to Reviewer**

**Reviewer #2 Comment 1**

The authors do not explain enough the concepts used in the paper like: Ambidextrous marketing, PLOR, PLOI, MC, ML, MO. The abbreviations are excesively used so that the readers beacome really confused.

**Response:**

We sincerely thank the reviewer for highlighting this important aspect. We fully understand that the overuse of abbreviations without adequate conceptual explanation can hinder readability, especially for readers who may not be familiar with these terms.

1. In response, we carefully and comprehensively reviewed the manuscript to ensure that all key concepts are clearly defined upon their first appearance. Specifically, we have provided explicit definitions for the following terms:

Ambidextrous Marketing (line 175-178; page 7), Marketing Exploration (line 178-180; page 7), Marketing Exploitation (line 180-182; page 7), Sustainable Marketing Capability (line 238-242; page 10), Marketing Culture (line 247-248; page 10), Marketing Learning (line 249-250; page 10), Marketing Operation (line 251-252; page 10), Corporate Performance (line 307-318; page12-13), Market Environment (line 382-384; page 15), and Policy Environment (line 385-388; page 15-16).

2. Regarding the use of abbreviations, we have revised the manuscript to follow a consistent and reader-friendly strategy. Specifically, abbreviations are applied primarily in sections where a term appears frequently and repetitively to improve clarity without compromising academic rigor. Our approach follows three main principles.

- In each major section (e.g., Introduction, Literature Review, Methodology), we spell out the full term with its abbreviation in parentheses at first mention. Thereafter, only the abbreviation is used in areas where the term occurs repeatedly.
- When discussing the theoretical foundations or measurement design, we consistently use the full term again to preserve academic precision and support reader comprehension.
- In all tables, abbreviations are retained to enhance layout clarity and avoid space constraints, particularly in wide-format ones.

To further facilitate transparency in the revision process, all locations where abbreviations were modified—whether full terms were added, abbreviations replaced, or adjustments were made for consistency—are clearly marked in **red font** in the revised manuscript.

We hope that this approach resolves your concerns and improves the manuscript’s readability. If further refinements are needed, we would appreciate your suggestions and would be happy to revise accordingly.

**Reviewer #2 – Comment 2**

2. The methodology is not enough explained. How the questionnaire looks like? The authors declare that the final sample includes 96 enterprises, but the data were collected from “352 valid questionnaires were retained from 47 companies”. Why the authors present the sample characterstics in the Results section?

**Response:**

Thank you very much for your detailed and constructive comments on our methodology and sampling. We sincerely appreciate the opportunity to clarify the following issues.

(1) Regarding the methodology, we have added a new subsection (Section 3.1 Research Design, line 436-445; page 18) at the beginning of the Methodology section. This section now clearly explains our overall research strategy: a quantitative approach using structural equation modeling as the primary analysis technique, supported by bootstrapping for mediation testing and latent moderated structural equations for moderation analysis. This aligns directly with our research questions.

(2) Regarding the questionnaire structure, we would like to clarify that the measurement items corresponding to each latent variable are already displayed in Table 3 under the “Variables and Items” column, which fully reflects the content of the questionnaire. The full version of the questionnaire was submitted as a supplementary material during the initial submission. If needed, we are happy to upload the questionnaire again, along with this revised manuscript, for your convenience.

(3) Regarding sample inconsistency, we apologize for the confusion caused by the earlier wording. The figure of 96 enterprises refers to the planned sample size, calculated based on Cochran’s formula and proportionally allocated across the provinces. However, due to constraints such as enterprise willingness and accessibility, valid responses were ultimately collected from 47 enterprises, resulting in 352 valid response questionnaires. We have revised Sections 3.4 Sampling (line 504-507; page 21) and 4.1 Sample Characteristics (line 524-530; page 22) to clearly distinguish between the planned and actual samples to avoid further ambiguity.

(4) Regarding the placement of the sample characteristics section, we agree that clarity of structure is important. The reason we presented Sample Characteristics under the Results section (Section 4.1 Sample Characteristics) is that these reflect the actual structure of the valid responses received, which could not be known in advance during the planning stage. In contrast, the sample information in the Methodology section (Section 3.4 Sampling) reflects the intended framework. We believe that this division is consistent with empirical research practice; however, if the reviewer suggests consolidation, we will be happy to make further adjustments.

Once again, we appreciate your thoughtful feedback, which has helped us improve the clarity and precision of the manuscript.

**Reviewer #2 – Comment 3**

Results. It is not very clear how the dimensions presented in Table 3 have been obtained? Were they designed from the beginning or the EFA was used?

**Response:**

Thank you for your question regarding the origin of the dimensions in Table 3. We would like to clarify that the measurement items and dimensions used in this study were derived from previously validated and widely accepted scales in the literature. Where necessary, we made minor adjustments to better reflect the research context of time-honored brands in southwestern China. This theoretical and literature-based approach was chosen to ensure content validity and comparability with previous studies.

To ensure reliability and validity, we conducted expert reviews and a pilot study before formal data collection. Subsequently, we applied confirmatory factor analysis (CFA) to verify the factor structure and assess the measurement quality of the model.

Therefore, we did not perform exploratory factor analysis (EFA) in this study. As this is a theory-driven research design, the measurement dimensions were conceptually and theoretically defined from the outset rather than derived from empirical data. This rationale is clearly explained in Section 3.3 Measurement (lines 456–462; page 19) of the revised manuscript. Additionally, to enhance clarity and transparency, we have carefully marked the source references for each measurement scale in **red font** within the 3.3 Measurement. We hope that this approach meets your expectations, and we would be grateful for any further suggestions you may have.

**Reviewer #2 – Comment 4**

**Reviewer Comment:**
In the Conclusion section the research questions are not addressed any more. The contribution of the research should be emphasized. The implications of the research results for theory and practice are not clearly described in the article. A detailed explanations of the author’s recommendations should be included

**Response:**

Thank you for your insightful and constructive suggestions. We fully agree that the conclusion section should clearly revisit the research questions and that the theoretical contributions, practical implications, and author recommendations should be explicitly addressed.

In response to your comments, we have made substantial structural and content enhancements to the manuscript.

- We have completely revised Section 5. Discussion and Section 6. Conclusion: To improve the clarity and coherence of the findings and to more effectively contextualize their significance.
- The responses to the research questions are now clearly and systematically addressed in Section 6. Conclusion, where each question is explicitly answered in light of our empirical results.
- The Theoretical Contributions of our study are summarized in detail in Section 5.4 Theoretical Contributions, highlighting how our research extends existing theories and models, particularly regarding ambidextrous marketing and sustainable marketing capability in traditional cultural enterprises.
- The Managerial Implications and actionable recommendations are thoroughly discussed in Section 5.3, Managerial Implications, offering specific guidance for business managers, heritage brand strategists, and policymakers.

These revisions were made with the utmost care to align with your expectations and enhance the academic and practical value of our study. We sincerely hope that these improvements address your concerns and strengthen the overall impact of the manuscript. We remain open to any suggestions you may have.

Additional Editorial Note Response – Figure Submission via PACE

In accordance with the journal’s requirements, we successfully uploaded all main manuscript figure files to the Preflight Analysis and Conversion Engine (PACE) digital diagnostic tool and confirmed that they met the technical standards set by PLOS.

We carefully reviewed and validated each figure for resolution, format, and layout compliance. All figures passed the diagnostic check, and the final versions have been included in our revised submission.

Should any further formatting needs or clarifications be required, we would be happy to address them promptly.

We sincerely appreciate the time and effort invested by the reviewers and the editorial team in providing constructive feedback on our manuscript. Their insightful suggestions have significantly contributed to enhancing the clarity, rigor, and overall quality of our work. We hope that the revised version satisfactorily addresses all the concerns and meets the standards for publication in PLOS ONE.

Please do not hesitate to contact us should any further information or clarification be required. We look forward to your favorable consideration.

Warm regards,

Dr. Li Xiangyu

Email: axgg2046@hotmail.com; axgg2046@outlook.com
